# Identifying Antibiotic Use Targets for the Management of Antibiotic Resistance Using an Extended-Spectrum β-Lactamase-Producing *Escherichia coli* Case: A Threshold Logistic Modeling Approach

**DOI:** 10.3390/antibiotics11081116

**Published:** 2022-08-17

**Authors:** Mamoon A. Aldeyab, Stuart E. Bond, Barbara R. Conway, Jade Lee-Milner, Jayanta B. Sarma, William J. Lattyak

**Affiliations:** 1 Department of Pharmacy, School of Applied Sciences, University of Huddersfield, Huddersfield HD1 3DH, UK; 2 Pharmacy Department, Mid Yorkshire Hospitals NHS Trust, Wakefield WF1 4DG, UK; 3 Institute of Skin Integrity and Infection Prevention, University of Huddersfield, Huddersfield HD1 3DH, UK; 4 Department of Microbiology, Mid Yorkshire Hospitals NHS Trust, Wakefield WF1 4DG, UK; 5 Scientific Computing Associates Corp., River Forest, IL 60305, USA

**Keywords:** antibiotic use, antibiotic resistance, antibiotic prescribing, antibiotic stewardship, threshold logistic modeling, thresholds, ESBL-producing *E. coli*, epidemiology, clinical practice

## Abstract

The aim of this study was to develop a logistic modeling concept to improve understanding of the relationship between antibiotic use thresholds and the incidence of resistant pathogens. A combined approach of nonlinear modeling and logistic regression, named threshold logistic, was used to identify thresholds and risk scores in hospital-level antibiotic use associated with hospital-level incidence rates of extended-spectrum β-lactamase (ESBL)-producing *Escherichia coli* (*E. coli*). Threshold logistic models identified thresholds for fluoroquinolones (61.1 DDD/1000 occupied bed days (OBD)) and third-generation cephalosporins (9.2 DDD/1000 OBD) to control hospital ESBL-producing *E. coli* incidence. The 60th percentile of ESBL-producing *E. coli* was determined as the cutoff for defining high incidence rates. Threshold logistic analysis showed that for every one-unit increase in fluoroquinolones and third-generation cephalosporins above 61.1 and 9.2 DDD/1000 OBD levels, the average odds of the ESBL-producing *E. coli* incidence rate being ≥60th percentile of historical levels increased by 4.5% and 12%, respectively. Threshold logistic models estimated the risk scores of exceeding the 60th percentile of a historical ESBL-producing *E. coli* incidence rate. Threshold logistic models can help hospitals in defining critical levels of antibiotic use and resistant pathogen incidence and provide targets for antibiotic consumption and a near real-time performance monitoring feedback system.

## 1. Introduction

The emergence and spread of multidrug-resistant bacteria pose a significant threat to public health and incur significant costs to healthcare systems and to society [1,2,3,4,5]. Studies have reported on the inappropriate use of antibiotics [6,7,8,9] and demonstrated a link between antibiotic use and the emergence and spread of antimicrobial resistance (AMR) globally [10,11,12,13,14,15,16,17,18,19]. Whereas antimicrobial stewardship informs the appropriateness of antibiotic use [7,9,20], balancing access to effective antimicrobials with the need to control AMR is challenging [12,13,19,21]. This requires a better understanding of the relationships between population antibiotic use and resistance [10,11,12,13,19]. Time series analysis using linear methods has been applied to measure the relationship between antibiotic use and resistance in ecological population studies [11,22,23,24,25,26,27]. However, nonlinear relationships between antibiotic use and resistance are suggested to be more likely by theoretical and mathematical models [10,12,13,19,28,29,30]. Studies suggest that there is a “threshold” level of a drug, above which the persistent selection of the antibiotic will lead to the development and spread of AMR [12,13]. These identified thresholds can be translated into targets for antibiotic use, thus, balancing access to therapy while controlling resistance [12,13].

While nonlinear time series analysis and other value segmentation modeling techniques have shown promise in identifying thresholds in antibiotic use associated with resistant pathogens, studies are needed to explore how increased antibiotic use above the identified thresholds relates to observed levels of resistance versus predicted levels. This study aims to develop a logistic modeling concept to improve understanding of the effect of antibiotic use on antibiotic resistance when usage exceeds recommended threshold levels, with the utility of providing targets for antibiotic consumption and providing a near real-time performance monitoring feedback system. For this study, considering data availability for analysis, extended-spectrum β-lactamase (ESBL)-producing *Escherichia coli* (*E. coli*) was selected to demonstrate the utility of the modeling concept. In addition, ESBL-producing *E. coli* is listed as an antibiotic-resistant pathogen for which new treatment regimens are urgently required [31].

## 2. Results

**Figure 1:**
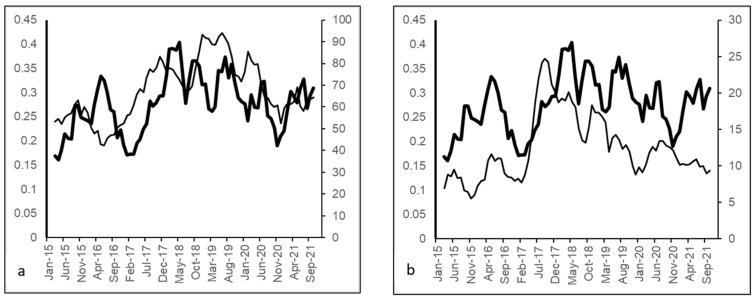
Monthly ESBL-producing *E. coli* incidence versus use of selected antibiotic classes (thick line, ESBL-producing *E. coli*, no. of cases/1000 OBD, 5-month moving averages, left-hand y-axis; thin line, antimicrobial use, DDD/1000 OBD, 5-month moving averages, right-hand y-axis): (**a**) fluoroquinolones; (**b**) third-generation cephalosporins.

Over the study period, 462 nonduplicated ESBL-producing *E. coli* cases were identified. The average monthly ESBL-producing *E. coli* incidence rate was 0.273 cases/1000 OBD (range: 0.051–0.543). Average fluoroquinolone use was 67.2 DDD/1000 OBD (range: 37.4–123.0), and average third-generation cephalosporin use was 12.3 DDD/1000 OBD (range: 3.4–31.3). A plot of the identified antibiotics and incidence of ESBL-producing *E. coli* cases is shown in Figure 1.

### 2.1. Defining a Critical Level of Pathogen Incidence Rates

**Figure 2:**
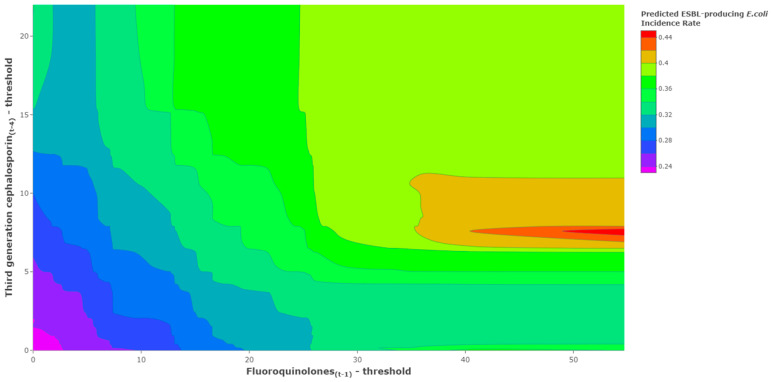
Illustrations of associations between antibiotic use above their identified thresholds and predicted ESBL-producing *E. coli* incidence rates.

Using continuous ESBL-producing *E. coli* incidence rates as the response variable, fluoroquinolones were found to have a 1-month lag relationship to ESBL-producing *E. coli* and a threshold of 71.0 DDD/1000 ODB (coefficient (95% CI) = 0.003 (0.001 to 0.006); *p* = 0.01). Third-generation cephalosporins were found to have a 4-month lag relationship to ESBL-producing *E. coli* and a threshold of 9.2 DDD/1000 ODB (coefficient (95% CI) = 0.005 (0.000 to 0.010); *p* = 0.04). The contour chart (Figure 2) shows the results of triangulating antibiotic unit changes above the identified threshold levels with the predicted ESBL-producing *E. coli* incidence rate. As the levels of antibiotic use increased, we observed increases in the predicted ESBL-producing *E. coli* incidence rate mapped by the transition of colors on the legend scale. There are sparse instances of fluoroquinolones above 40 DDD/1000 OBD above its threshold and no instances of both fluoroquinolones above 40 DDD/1000 OBD coinciding with third-generation cephalosporins above 12 DDD/1000 OBD above its threshold. The x-axis is the threshold-adjusted fluoroquinolone use at lag 1, and the y-axis is the threshold-adjusted third-generation cephalosporin use at lag 4. The lower-left corner of the plot is the point at which both antibiotic series are equal to their identified threshold levels; third-generation cephalosporins (t − 4) = 9.2 DDD/1000 OBD and fluoroquinolones (t − 1) = 71.0 DDD/1000 OBD, which translates into 0 values for the model components or basis functions. 

The basis functions are:

$$Threshold\;and\;lag\;adjusted\;third\;generation\;cephalosporin\;=max\left(Third\;generation\;cephalosporin{\;}_{\left(t-4\right)}-9.2,\;0\right)$$

$$Threshold\;and\;lag\;adjusted\;fluoroquinolone=max\left(fluoroquinolone_{\left(t-1\right)}-71.0,\;0\right)$$

The information gained from the above threshold model on the continuous ESBL-producing *E. coli* incidence rates, such as lag relationships and candidate antibiotic threshold values, was then exported into a threshold logistic regression search algorithm to identify the cutoff percentile value of ESBL-producing *E. coli* that we established as a high incidence rate or critical level. The Akaike information criterion (AIC) was used to select the 60th percentile of ESBL-producing *E. coli* as the critical level to define the binary dependent variable for logistic modeling. It is this cutoff percentile that resulted in the greatest separation of the binary class (1 = exceeding cutoff; 0 = below cutoff) and best classification accuracy.

### 2.2. Threshold Logistic Method

**Figure 3:**
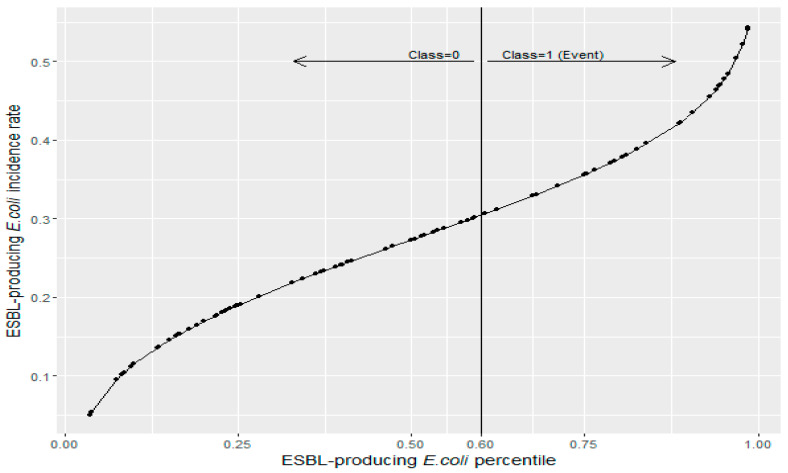
The empirical cumulative distribution function for ESBL-producing *E. coli* historical data. The solid vertical line represents the 60th percentile (0.288 cases/1000 OBD).

The empirical cumulative distribution function for ESBL-producing *E. coli* historical data is presented in Figure 3. The 60th percentile (0.288 cases/1000 OBD) of the ESBL-producing *E. coli* incidence rate was selected as the cutoff in defining the dichotomous binary classification variable (Figure 3) based on the search results.

A summary of the final threshold logistic model is provided in Table 1.

**Figure 4:**
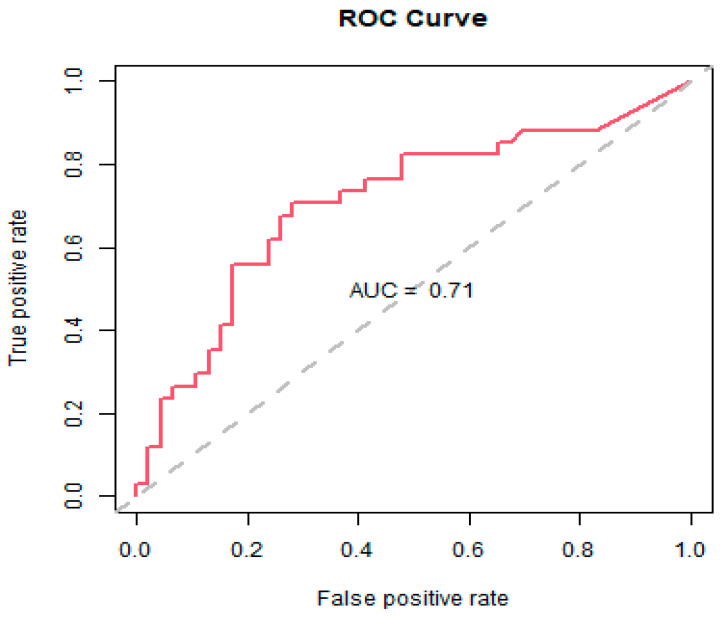
Receiver operator characteristic (ROC) chart plots the true positive classification rate against the false positive classification rate at different probability cutoff thresholds. The area under the curve (AUC) is an aggregate measure of performance across all possible classification thresholds.

**Figure 5:**
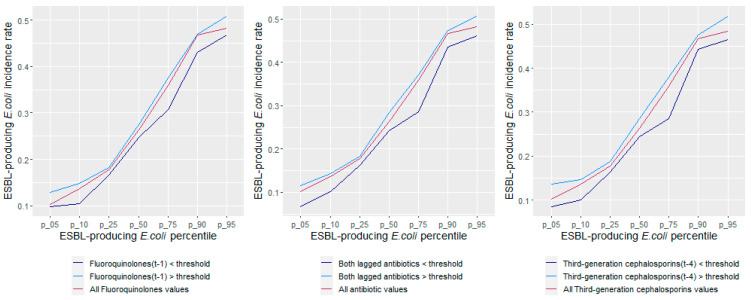
The cumulative ESBL-producing *E. coli* incidence rates relative to fluoroquinolone and third-generation cephalosporin use being above or below their respective thresholds.

The introduced variable representing the COVID-19 period was not found to be significant (coefficient (95% CI) = 0.252 (−0.810 to 1.314); *p* = 0.642) and was removed from the models to maintain parsimony. The threshold logistic model showed that for every one-unit increase in fluoroquinolones and third-generation cephalosporins above 61.14 and 9.16 DDD/1000 OBD, the average odds of the ESBL-producing *E. coli* incidence rate being ≥60th percentile of historical levels increased by 4.5% and 11.9%, respectively. The model produced a 70% classification accuracy and an AUC measure of 71% for the ROC curve (Figure 4). Figure 5 displays the cumulative ESBL-producing *E. coli* incidence rates relative to fluoroquinolones and third-generation cephalosporins being above or below their respective thresholds. ESBL-producing *E. coli* incidence rates were consistently higher when the antibiotic thresholds were exceeded and consistently lower when the antibiotic thresholds were kept below their defined thresholds.

**Figure 6:**
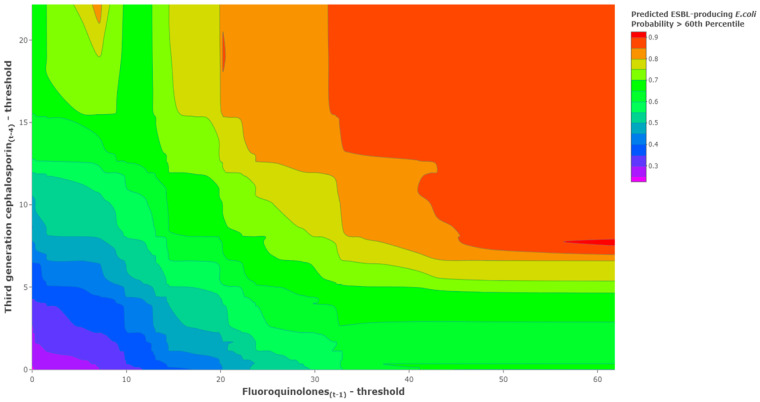
Results of triangulating antibiotic unit changes above identified threshold levels and the predicted probability of exceeding the 60th percentile of historical ESBL-producing *E. coli* using the identified threshold logistic model.

As a means of comparison to the threshold model based on the continuous ESBL-producing *E. coli* incidence series, a contour plot was produced using the threshold logistic model (presented in Table 1) but now shows the predicted probability of exceeding the 60th percentile of the historical ESBL-producing *E. coli* incidence rate (Figure 6). Probabilities ranged from near zero when antibiotics were close to their thresholds to approaching near certain probability (≥99%) as antibiotic use increased above their thresholds. The x-axis is threshold-adjusted fluoroquinolone use at lag 1, and the y-axis is threshold-adjusted third-generation cephalosporin use at lag 4. The lower-left corner of the plot is the point at which both antibiotic series are equal to their identified threshold levels: third-generation cephalosporins (*t* − 4) = 9.16 DDD/1000 OBD and fluoroquinolones (t − 1) = 61.14 DDD/1000 OBD. It is the point at which the basis functions evaluate to 0.

The basis functions are:

$$Threshold\;and\;lag\;adjusted\;third\;generation\;cephalosporin\;=max\left(Third\;generation\;cephalosporin{\;}_{\left(t-4\right)}-9.16,\;0\right)$$

$$Threshold\;and\;lag\;adjusted\;fluoroquinolone=max\left(fluoroquinolone_{\left(t-1\right)}-61.14,\;0\right)$$

As the combined levels of antibiotic use increased, we observed increases in the probabilities of reaching the established high incidence rate of ESBL-producing *E. coli*.

### 2.3. Risk Scores

The risk scores produced from the threshold logistic model for ESBL-producing *E. coli* incidence rates exceeding the 60th percentile (0.288 cases/1000 OBD) are shown in Table 2. The probability risk scores were then coded into three alert signal levels (low–medium–high), which are also summarized in Table 2.

As a guide to understanding the table, we observe the row of information for January 2021. The ESBL-producing *E. coli* incidence rate was below 0.288 (60th percentile) in January 2021. Fluoroquinolone use (DDD/1000 OBD) was below 61.14 in the previous month, which recodes the basis function for fluoroquinolone use to 0. Third-generation cephalosporin use (DDD/1000 OBD) was above 9.16 4 months previous, which recodes the basis function for third-generation cephalosporin use to 5.07. Applying these values to the threshold logistic model, a predicted probability of 0.3472 is produced, which translates into a medium alert signal. The predicted ESBL-producing *E. coli* rate (0.2595) was produced using the threshold model identified for the continuous ESBL-producing *E. coli* rate along with one standard deviation above the predicted value (0.2799), both of which are below the 0.288 (60th percentile).

Table 3 summarizes the overall classification accuracy based on the coded alert signals that stem from the threshold logistic model. We found that a low alert signal was correct 14 out of 19 times to identify the ESBL-producing *E. coli* incidence rate below the 60th percentile (a 2.8 to 1 accuracy ratio). A high alert signal was correct 17 out of 25 times in identifying the ESBL-producing *E. coli* incidence rate above the 60th percentile (a 2.1 to 1 accuracy ratio). For the medium alert signal, we were twice as likely to be below the 60th percentile of the ESBL-producing *E. coli* incidence rate.

### 2.4. What-If Scenarios

In addition to being able to report on ongoing performance, if the predictive model possessed a lag structure, the model could be used to perform a “what-if” scenario by adjusting expected antibiotic levels that influence future ESBL-producing *E. coli* incidence rates. In the model presented here, third-generation cephalosporins enter the model 4 months prior to the current month, and fluoroquinolones enter the model 1 month prior to the current month. Consequently, what-if scenarios could be used to predict the probability of ESBL-producing *E. coli* incidence rates above the 60th percentile 3 months ahead based on the observed levels of third-generation cephalosporins and testing values for fluoroquinolones, as shown in Table 4.

The last three rows of the table are future months in which we can obtain third-generation cephalosporin use. The predicted levels and probabilities for these future months were based on undefined fluoroquinolone use and, thus, equivalent to being below its 61.14 DDD/1000 OBD threshold. By substituting a range of possible fluoroquinolone values in the gray highlighted cells, the resulting predicted levels and probabilities for the ESBL-producing *E. coli* incidence rate can be examined. It would be advantageous to ensure fluoroquinolone use is constrained as much as possible and kept below 61.14 DDD/1000 OBD, especially for February 2022 and April 2022, so as not to elevate the alert above medium (Table 4).

## 3. Discussion

This study demonstrated the development and utility of an innovative modeling concept called threshold logistic modeling, which employs a combined approach of MARS and logistic regression. Using the threshold logistic approach, we addressed the following: determining the cutoff percentile value of ESBL-producing *E. coli* that defines a high incidence rate, identifying thresholds for fluoroquinolones and third-generation cephalosporins to control ESBL-producing *E. coli* incidence rates in hospitals, improving our understanding of the effect of antibiotic use on the probability of reaching a specified critical level of pathogen incidence when antibiotic usage exceeds recommended threshold levels, providing risk scores of an event exceeding critical levels, and providing a near real-time performance monitoring feedback system through a scorecard approach.

Threshold modeling (e.g., MARS) has been exclusively employed on a continuous response variable such as the incidence rate of a specific pathogen in hospitalized patients [12,13]. A key aspect of threshold modeling for antimicrobial resistance concentrates on the threshold values since antibiotic policy attempts to curtail antibiotic use from exceeding those identified thresholds in a given period [12,13]. The threshold value is the start of a measurable difference in the response function when compared with the remaining segment of the explanatory variable. While the threshold value is the start of an observed increase in the incidence rate of a specific pathogen, it is not indicative of the start of the incidence rate that is outside normal variation. However, increased levels of antibiotic use that are progressively above the threshold are associated with an increased probability of incidence rates outside normal variation. This was shown in the contour plots (Figure 2), which illustrated an increase in predicted ESBL-producing *E. coli* incidence rates as levels of antibiotic use increased. Consideration of the full model helped to provide more information on the expected magnitude of the impact of using antibiotics on the incidence of hospital pathogen rates. These observations highlighted the need to define a critical level of pathogen incidence rates along with predicting the probability of reaching it. Using the threshold logistic approach, we identified and confirmed the 60th percentile in ESBL-producing *E. coli* as the cutoff level that defines a high incidence rate, and we produced a predicted probability of exceeding the 60th percentile of a historical ESBL-producing *E. coli* incidence rate (Figure 6). The threshold logistic model identified thresholds for fluoroquinolones and third-generation cephalosporins that would accelerate ESBL-producing *E. coli* incidence rates and increase the probability of reaching critical levels.

Maintaining antibiotic use within small tolerances of the thresholds will control ESBL-producing *E. coli* incidence rates and maintain rates below critical levels. Over time, average ESBL-producing *E. coli* incidence rates should naturally fall as future months of pathogen incidence are realized below the ESBL-producing *E. coli* 60th percentile. With an understanding that we are using modeling techniques to affect future pathogen incidence rates, threshold models should be retrained on a periodic basis to adjust to the evolutionary changes of the data. The contour plot provided a valuable tool to understand the risk of antibiotic use above the stated thresholds and the reward of maintaining a specific policy.

The utility of logistic regression is in its ability to provide a risk score or probability of an occurring event in terms of a set of covariates taken together. Because the probability score is theoretically bounded by a minimum probability value of 0 and a maximum probability value of 1, it is a measure that can be used across all policy implementations without regard to the scale of the pathogen incidence rate. Whereas the basic idea remains valuable—to provide guidance to hospitals on keeping individual antibiotics usage below certain threshold levels—it is essential to also consider the overall model(s) from which the individual thresholds were derived to monitor predictive performance and to be alert to potential increases in pathogen incidence rates for the coming month through the predicted values of the overall model, which may include both the predicted level of the pathogen incidence rate as well as a risk score or probability of the pathogen incidence rate exceeding a critical level. In this study, a sample scorecard was created to blend these ideas together and offered (a) an ability to obtain near real-time feedback for policy assessment and for keeping antibiotic use below identified thresholds, (b) an ability to identify breakdowns in model performance due to changes in the environment or relationship of antibiotic usage levels to antimicrobial resistance of the pathogen, and (c) an opportunity to perform “what-if scenarios” using varied levels of antibiotic use to assess the expected impact on risk scores and predicted pathogen incidence rate for upcoming months.

This study has the strength of employing rigorous analysis methods and utilizing routinely collected data for all hospitalized adult patients; therefore, selection and information bias are unlikely. However, since the study was at the hospital population level, it was not possible to adjust for potential changes in the patient population, and the case mix and present model could have been improved via the inclusion of further explanatory variables if this were possible. Examples of further explanatory variables data include infection prevention and control activities and proxy measures for changes in the patient population and case mix [32,33]. Finally, this work represented a single-center assessment; therefore, the study would benefit from a multi-center assessment.

In conclusion, using routinely generated data, threshold logistic models determined the thresholds for fluoroquinolones and third-generation cephalosporins in relationship to ESBL-producing *E. coli* incidence rates in hospitalized patients. These thresholds can inform better hospital antibiotic stewardship, avoiding the challenge of implementing a complete restriction of antibiotics in clinical practice [21] and maintaining the diversity of prescribing. Threshold logistic models can help hospitals in defining a critical level of pathogen incidence rates and provide risk scores of an event reaching critical levels. The latter estimated risk scores can be utilized to create an alert signal and provide performance monitoring feedback. We also look to this new method as complementary to the standard methods that are currently in place for antimicrobial stewardship [12,13]. As such, both modeling concepts provide different ways to investigate the data and, combined, produce a robust set of metrics that can be used to develop a systematic approach to control antimicrobial resistance. Further work is needed to test the impact of this approach in informing antimicrobial stewardship and controlling resistance rates in hospitals.

## 4. Methods

### 4.1. Study Design and Population

The study was undertaken at Pinderfields Hospital (700 beds), Mid Yorkshire Hospitals NHS Trust in West Yorkshire, England. The Trust cares for 500,000 people, providing medical and surgical services, intensive care, hematology/oncology, regional burns, regional spinal injuries, and community services. This study included collecting retrospective data from January 2015 to December 2021. The study population included all adult inpatients admitted to Pinderfields Hospital. For this analysis, the minimum requirement was 5 years of monthly antibiotic use and microbiology data (60 monthly observations) [12,13]. This study used 7 years of data and was determined based on the availability of the longest period of consistent explanatory (antibiotic use) and outcome (ESBL-producing *E. coli*) variables data.

For the purpose of this study, it was hypothesized that the use of fluoroquinolones and third-generation cephalosporins could explain variations in the incidence of ESBL-producing *E. coli*. These antibiotics were identified a priori based on their resistance profiles obtained from the hospital microbiology department (which showed that ESBL-producing *E. coli* isolates were resistant to cefpodoxime and ciprofloxacin in 100% and 76.8% of the cases, respectively) and published evidence of their role as risk factors for driving ESBL-producing *E. coli* incidence rates in hospitals [12,15,25].

### 4.2. Microbiology and Pharmacy Data

Infection control software (ICNET) was interrogated for blood and urine cultures. Adult inpatients (≥18 years) who had a positive ESBL-producing *E. coli* result while admitted to Pinderfields Hospital between 01/01/2015 and 31/12/2021 were defined as an ESBL-producing *E. coli* case. Any duplicates were excluded if they were within 30 days of hospital readmittance. The isolates were screened firstly using a cefpodoxime disk. If resistant, they were tested for ESBL using a “double-disk synergy test.” ESBL-producing *E. coli* were identified according to standard microbiological procedures in line with the European Committee on Antimicrobial Susceptibility Testing (EUCAST) guidelines [34]. Monthly antibiotic use data were obtained from the hospital pharmacy information systems (JAC). These data were then converted into defined daily doses (DDD) and expressed as DDD per 1000 occupied bed days (OBD). The DDD was calculated according to the classification of antimicrobials for systemic use (J01) in the WHO/ATC index [35]. We calculated the number of DDD for each antibiotic substance by dividing the used amount in grams of the antibiotic substance by the assigned DDD value to that substance (i.e., number of DDDs = grams of active substance/substance-specific DDD) [35,36].

### 4.3. Modeling and Statistical Analysis

Analysis started with producing descriptive statistics and plotting the antibiotic series alongside the ESBL-producing *E. coli* incidence rates. This was followed by examining the cross-correlation functions of the series to better understand the lag structure and relationship. We then considered that nonlinear relationships might be present in the data and explored the possibility that thresholds could exist in antibiotic use that altered ESBL-producing *E. coli* incidence rates. We initially applied general additive models (GAMs), but we used multivariate adaptive regression spline (MARS) methods and other value-segmenting models to detect threshold values and lagged relationships [37,38,39,40]. In order to use a logistic approach, the continuous pathogen rate must be converted into a binary event [41]. The binary event was defined as a critical level of pathogen incidence rate that is set by clinical mandate and (or) through exploratory numerical methods.

#### 4.3.1. Defining a Critical Level of Pathogen Incidence Rate

MARS and nonlinear value segmentation threshold methods were initially used to model the continuous pathogen incidence rates relative to the antibiotic variables. We also included a binary indicator variable to evaluate any shift that might have occurred due to COVID-19 beginning in March 2020. The structure of the threshold model that best described the relationship between antibiotic use and the continuous pathogen incidence rate was used to identify the initial critical level of incidence rate that produced the highest classification accuracy using a search algorithm. The search algorithm used the increasing percentile values of pathogen incidence rates to transform the continuous pathogen incidence rate into a binary event. The binary event took the value of 1 if the pathogen incidence rate exceeded the evaluated percentile; 0 otherwise. The search algorithm estimated the threshold logistic model at each iteration to measure both goodness of fit and classification accuracy. The search was constrained between the 50th and 85th percentile of pathogen incidence rates, as it is a target that is in the upper 50% of the historical data and includes enough observations in the target range so as not to be a rare event. In our study, the 60th percentile (0.288) was identified as the cutoff, rendering the binary event as:

Event = Ifelse (ESBL-producing *E. coli* incidence rate ≥ 0.288, 1, 0) (1)

#### 4.3.2. Threshold Logistic Method

We refined the threshold model that was identified on the continuous pathogen incidence rate. MARS methods were applied by substituting the continuous ESBL-producing *E. coli* incidence rate with the binary event, as described above, for the dependent variable. The MARS coefficients were converted into logistic form in order to obtain predicted probabilities and odds ratios.

We also applied an alternative logistic search algorithm that jointly optimized the critical level, antibiotic threshold values, and lag structure of the antibiotic series in relation to the binary event. Although both the MARS algorithm and the threshold logistic search algorithm produced feasible models, the model produced from the threshold logistic search algorithm was best suited to policy adoption in that antibiotic thresholds were both left-truncated (i.e., the threshold-adjusted antibiotic series was coded to 0 if less than the threshold). The MARS model produced a right-truncated threshold in which the threshold-adjusted third-generation cephalosporin use had a declining slope for values continually less than the threshold.

The optimization algorithm applied robust logistic regression estimation [42,43]. Model selection was based on classification accuracy using a probability cutoff that maximized the sum of sensitivity and specificity to address class imbalance. The computed area under the curve (AUC) from the receiver operator characteristic (ROC) curve was also used as a confirmatory measure of classification power [44,45]. Upon optimizing the threshold breakpoints, a sensitivity analysis was conducted on the lower and upper limit around the threshold value using a one-at-a-time (OAT) approach. We note that antibiotic thresholds were refined using the threshold logistic search algorithm. The overall lag structure remained consistent with the continuous approach, and the critical level was confirmed to be of the highest accuracy between the 55th and 60th percentile, with the 60th percentile being dominant and optimal.

Predicted probabilities of the threshold logistic regression model were produced, which are synonymous with the risk scores. We also created a coded alert signal (high–medium–low risk) that was based on the MinMax transformation of the predicted probabilities (risk score) coming from the threshold logistic model.

(2) $$z=\frac{prob-\min\left(prob\right)}{\max\left(prob\right)-\min\left(prob\right)}$$

The cutoff ranks were computed using a linear programming (LP) technique to maximize the overall distribution accuracy of a low signal classifying an infection rate below the 60th percentile and a high signal classifying an infection rate greater than the 60th percentile. The cutoff ranks optimized through LP were 0.0 to ≤0.24, >0.24 to <0.70, and ≥0.70 to 1.0 to define low–medium–high coded alert signals, respectively.

Analysis was performed using the SCA Statistical System version 8.2 (Scientific Computing Associates Corp., River Forest, IL, USA) and R software (R Foundation for Statistical Computing, Vienna, Austria).

## Figures and Tables

**Table 1:** Threshold logistic result in modeling ESBL-producing *E. coli* incidence rates at 60th percentile, January 2014 to December 2021.

Predictor Variable	Lag	Median Use (IQR)	Threshold (95% Confidence Limit) *	Relation to Threshold	Coefficient (95% CI)	*p*-Value	Odds Ratio (95% CI)
Constant	NA	NA	NA	NA	−1.177 (−1.902 to −0.451)	0.0034	0.279 (0.119 to 0.656)
Fluoroquinolone use (DDD/1000 OBD)	1	64.55 (53.58–76.39)	61.142 (55.96 to 68.27)	Above	0.045 (0.005 to 0.085)	0.0293	1.045 (1.004 to 1.088)
Third-generation cephalosporin use (DDD/1000 OBD)	4	10.76 (7.90–14.9)	9.159 (8.75 to 11.40)	Above	0.108 (0.004 to 0.211)	0.0414	1.119 (1.007 to 1.244)

* 95% confidence limit around the optimized threshold value, which was derived using a one-at-a-time (OAT) approach; IQR, interquartile range; NA, not applicable.

**Table 2:** Risk scores for ESBL-producing *E. coli* incidence rate exceeding the 60th percentile for 2021.

Date	ESBL-Producing *E. coli* Observed Above 60th Percentile (0.288)	Fluoroquinolone Use (DDD/1000 OBD) at Lag 1 (Threshold-Adjusted)	Third-Generation Cephalosporin Use (DDD/1000 OBD) at Lag 4 (Threshold-Adjusted)	Predicted ESBL-Producing *E. coli* Level *	Predicted ESBL-Producing *E. coli* Level + 1 Standard Deviation (SD) *	Predicted Probability ESBL-Producing *E. coli* >60th Percentile	Coded Alert Signal
January	Below	0.00	5.07	0.2595	0.2799	0.3472	Medium
February	Below	0.00	5.52	0.2620	0.2823	0.3585	Medium
March	Below	0.00	1.11	0.2386	0.2589	0.2578	Medium
April	Above	32.22	1.12	0.3142	0.3346	0.5949	High
May	Above	7.70	2.24	0.2446	0.2649	0.3563	Medium
June	Below	0.00	0.00	0.2327	0.2530	0.2356	Low
July	Below	0.00	0.82	0.2370	0.2574	0.2519	Medium
August	Above	10.07	1.71	0.2424	0.2627	0.3675	Medium
September	Above	0.00	0.57	0.2357	0.2561	0.2469	Medium
October	Below	0.00	2.65	0.2467	0.2671	0.2908	Medium
November	Below	12.62	1.54	0.2501	0.2705	0.3900	Medium
December	Below	5.26	2.23	0.2445	0.2649	0.3316	Medium

* Prediction was produced using the threshold model identified for the continuous ESBL-producing *E. coli* rate.

**Table 3:** Summary of numbers of coded alert signals when ESBL-producing *E. coli* observed above and below the 60th percentile (January 2015–December 2021).

		ESBL-Producing *E. coli* Incidence Rate Observed Above and Below the 60th Percentile	
		Above	Below
Coded Alert Signal	Low (<0.24)	5	14 (2.8:1)
	Medium	12	24
	High (>0.70)	17 (2.1:1)	8

**Table 4:** What-if threshold logistic model exploration.

Date	Fluoroquinolone Use (DDD/1000 OBD) at Lag 1 *	Third-Generation Cephalosporin Use (DDD/1000 OBD) at Lag 4	Predicted ESBL-Producing *E. coli* Level **	Predicted ESBL-Producing *E. coli* Level + 1 Standard Deviation (SD) **	Predicted Probability ESBL-Producing *E. coli* > 60th Percentile	Coded Alert Signal
January 2022	75.15 ↑	5.82 ↓	0.2467	0.2670	0.3658	Medium
February 2022	**0.00**	10.28 ↑	0.2386	0.2589	0.2580	Medium
March 2022	**0.00**	6.43 ↓	0.2327	0.2530	0.2356	Low
April 2022	**0.00**	12.83 ↑	0.2521	0.2725	0.3140	Medium

↑ Above threshold; ↓ below threshold; * The predicted levels and probabilities for February-April months were based on undefined fluoroquinolone use and, thus, equivalent to being below its 61.14 DDD/1000 OBD threshold; ** Prediction was produced using the threshold model identified for the continuous ESBL-producing *E. coli* rate.

## Data Availability

The data is contained in the article.
